# The use of ayurvedic medicine in the context of health promotion – a mixed methods case study of an ayurvedic centre in Sweden

**DOI:** 10.1186/s12906-016-1042-z

**Published:** 2016-02-17

**Authors:** Maria Niemi, Göran Ståhle

**Affiliations:** The Integrative Care Science Center, Ytterjärna, 15391 Järna, Sweden; Karolinska Institutet, Department of Public Health Science, Tomtebodavägen 18 A, 171 65 Stockholm, Sweden; Södertörn University, School of Historical and Contemporary Studies, Alfred Nobels Allée 7, 141 89 Huddinge, Sweden

**Keywords:** Ayurveda, Sweden, Case study, Qualitative study, Pulse diagnosis

## Abstract

**Background:**

Ayurveda has its historical roots in India, but has also been internationalised, partly via migration and partly through an increased interest in alternative medicine in the West, where studies point toward increased use. However, there is to date scarce knowledge about the use and experiences of ayurveda in Sweden.

**Methods:**

We have conducted a case study of a center for ayurvedic healthcare in Sweden. We have collected information on client background data from the center’s documentation, and compiled data from all clients who visited the centre for ayurvedic consultation during spring 2014. In total, 55 individuals were included in the study, and 18 of them were chosen for individual semi-structured interviews, to gain a deeper understanding of their motives for seeking, and experiences of ayurvedic health care. The material was analysed and compiled through a mix of qualitative and quantitative methods.

**Results:**

Among the 55 clients, 91 % were female the mean age was 47 years, and 64 % gave a specific illness as a reason for seeking ayurveda. The most common illnesses were respiratory, musculoskeletal, circulatory, tumor, and cutaneous illnesses. The qualitative results showed that ayurveda was being used in combination with other methods, including various diets, other alternative medicine methods and conventional medicine. Some participants recounted having sought ayurveda as a complement to conventional medicine, or in cases when conventional medicine had been experienced as insufficient in terms of diagnosis or treatment. However, some participants experienced it as difficult to follow the ayurvedic life-style advice in the midst of their everyday life. Many participants reported positive experiences of pulse diagnostics, which was the main diagnostic method used in ayurvedic consultation. Some reported concrete, physical improvement of their symptoms.

**Conclusions:**

This study points towards important aspects of participant experience of ayurveda, that may be subject to further research. The positive effects experienced by some clients should be studied more systematically in order to discern whether they are specific or non-specific. In addition, interesting knowledge may be gained through further study of the reported positive experiences of pulse diagnosis.

## Background

Modern Ayurveda is a complex phenomenon that is both practiced as a whole system of medicine, and as various forms of self-care, including ayurvedic massage, diet, yoga, etc. Ayurveda has its historical roots in South Asia, but its present expressions are a product of globalisation, partly through migration and a growing interest in alternative health practices in the Euro-American context [1]. The present study focuses on the patterns of Ayurveda use in Sweden. Our approach is explorative, since there is a lack of previous research in the Swedish context. this is remarkable from an international perspective, since Ayurveda is one of the largest expressions of Complementary and Alternative Medicine (CAM) globally [2].

## Why study Ayurveda in the Swedish context?

The treatment of chronic illnesses that overwhelm both health-care and financial systems are great present day challenges [3], and finding new ways of fostering personal health and well-being. Modern biomedicine, while wildly successful in some areas, cannot fully address the growing epidemics of chronic illnesses [4]. Empirical and evidence based research on Ayurveda may offer useful knowledge as a complement to biomedicine, that can be used in integrative care, as Ayurveda pays attention to nutrition, daily routines, exercise, and mental harmony. Also, Ayurveda’s pharmacology, which is based on natural products, may offer an effective and well-tolerated means to managing certain diseases [5]. The World Health Organization has urged that the safe and effective use of traditional medicine by regulating, researching and integrating traditional medicine products, practitioners and practice into health systems, be promoted where appropriate [6]. Yet, scientific publications on Ayurveda in international peer reviewed journals have remained dismal [7]. Though a wealth of research has been conducted in South Asia, many of those clinical trials are not available in easily accessible journals, and some do not meet rigorous research standards [8].

Ayurveda relies on humoral diagnostics and therapeutics including a combination of internal medicine, herbal remedies, and rejuvenation programs [9]. Practitioners of Ayurveda point out that primary, secondary, and tertiary prevention, patient self-empowerment, and self-efficacy play crucial roles in the Ayurvedic approach to healing (reviewed in [10]). It is also often claimed that Ayurveda employs a personalised, multi-factorial approach to health care and cure, as opposed to the generalized and single target strategy of biomedicine [7].

## Multiple medical realities

In the Euro-American context, Ayurveda falls under the broad umbrella of CAM. Studies show an increasing number of patients seeking CAM, but in general there is a lack of government regulation regardsing standardisation of practice. These therapies are also not routinely offered in medical schools or graduate medical education [3]. With regard to Ayurveda in particular, it has spread mainly in Britain, the USA and Canada. In Germany, Austria, and Switzerland it is also one of the fastest growing CAM methods (reviewed in [10]). In general, there are no standards of required compliance for Ayurvedic colleges, each of which has its own guidelines for training and certificate programs [11]. In contrast, in contemporary India, Ayurveda is central to health provision nation wide. The Indian government has various acts and councils in place to regulate and control the practice of Ayurveda, provide professional training, and standardise education and qualifications. Ayurvedic institutions and colleges in India are based on the institutional and organizational model provided by biomedicine, and Ayurveda has thus become biomedicalised [12]. The BAMS (Bachelor in Ayurvedic Medical Sience) curriculum is organised primarily around a modern division of subjects rather than around particular Ayurvedic texts or topics [13].

Even if the national contexts are quite specific, it is also important to recognize that these local expressions of Ayurveda are interrelated in the globalization of Ayurveda. There is a considerable exchange between the Indian and Euroamerican contexts [1]. For example, Swedish entrepreneurs organize visits at health centres located in India that offer Ayurvedic purification treatments (pancha karma). Also, Indian trained Ayurvedic doctors are engaged in courses and workshops in Sweden.

To this date there is no systematic research about the use of Ayurveda in the Swedish context and more research in this area is needed. Probably, a large proportion of the Swedish population come into contact with different aspects of Ayurvedic treatments and products as part of self-care. In contrast, there are not many Indian trained Ayurvedic doctors in Sweden, as the population of South Asian immigrants is quite small compared to other European countries. However, the use of Ayurveda can be expected to be increasingly popular in conjunction with the general growth in popularity of CAM in the country [14].

There is no systematic information about the availability and diversity of Ayurvedic health-care education in Sweden. Licensed health care practitioners are not legally permitted to practice CAM as part of their professional vocation in the country, and alternative medical practices are regulated in the national health care legislation. For example, it is forbidden to treat children below 8 years of age and pregnant women. It is also forbidden to treat illnesses including diabetes, epilepsy, cancer, as well as various communicable diseases [15].

## Modern and global Ayurveda

Ayurveda is based upon practical knowledge that has been systematized and developed for long periods of time in South Asia. Treatises that date from the centuries before the Common Era are often referred to as paradigmatic [16].

However, the present expressions of Ayurveda are a product of modernization and globalization. The contact with Western colonialism initiated a process of modernization. This resulted in aspirations to systematize and unify the various indigenous South Asian traditions associated with Ayurveda, in order to become a viable complement to biomedicine as a whole system of medicine. One of the transformations was the standardization of medicaments in accordance with the principles of mass production [12]. This trend has been further accelerated through globalization and the incorporation of different aspects of Ayurveda in the health and beauty industry in the Euro-American context [12]. Another feature of the modernization processes is that Ayurveda in South Asia has tended to become increasingly secularized, in the sense that many of its spiritual and metaphysical connotations have been disregarded. Meanwhile, Ayurveda in the Euro-American context shows the opposite trend – of becoming sacralised or re-enchanted in relation to the ‘holistic milieu’ of ‘inner’ or ‘self’ spirituality [11]. This includes the products of the movements related to charismatic spiritual figures like Deepak Chopra and Sri Ravi Shankar. The association of globalized Ayurveda with the Euro-American self-care culture implies a trend in downplaying the role of medication and emphasizing life style advice [11]. This includes actively taking different courses, therapies and diets, but also actively reading books and educating oneself.

## Methods

### The aims and strategy of the study

To this date there are few studies on the participants in CAM-treatment in Sweden. The majority of previous research focuses on the therapists’ perspectives or the specific causal relationships of specific therapeutical variables on specific health parameters. As a contrast, the focus of this study is on the experiences and motives of the clients in Ayurvedic health counselling, and the related psychosocial processes and context. Therefore, a case study approach was used. A case study focuses on one expression of a naturally occuring phenomenon in order to gain a detailed description of experiences and/or processes in the particular case. By studying the single case, insights can be drawn that could have wider consequences - insights that would not have been found in another way [17]. The aim of this study is to develop knowledge of the clients’ perspectives on Ayurveda, through giving detailed descriptions of characteristics and nuances, and to analyze according to a systematic/critical method of reflection. We will also discuss the implications of these findings for further empirical medical research. The results of the study will thus give important contributions to understanding the use of CAM and holistic medicine in Sweden.

This research strategy implies that the present study does not take a stand on the effectiveness and safety of Ayurvedic treatments or medicaments, nor on the truth claims of the Ayurvedic philosophy. The focus is rather upon how this instance of CAM is used in this specific context and upon experiences of those who engage in it. The experienced results of the treatments are still of interest, but the focus is on the participants’ subjective experience of these results.

The specific aims of the study are summarized as:

- To develop descriptions of the clients’ experiences and motives for seeking Ayurvedic health counselling.
- To specify the key questions described by the participants, including nuances, differences, and complexity
- To compare the information that is gathered from the case with previous research about CAM in the Euro-American context, in order to learn from differences and similarities

### Why the unit of study was chosen

According to case study methodology, the case is selected against the background of its attributes and characteristics, not by the principle of random selection.

The present study focuses one of the major centres of Ayurveda in Sweden. The centre hosts treatments including yoga, massage, health counselling, rejuvenation (pancha karma), retreats as well as education programs. The centre has clear procedures for treatment and routines for client documentation. Therefore, this centre can provide access to a documented network of clients that is unique in CAM research and holistic health in Sweden. A centre like this attracts a large variety of persons who are engaged in different aspects of holistic health. This guarantees a width in the selection of participants for the research project.

Among the many different methods that are practiced at the present centre, Ayurvedic health counselling was chosen as the object of study. The main reason for this was that this is the most common form of treatment/councelling that is provided at the centre, and that it includes all the main components of Ayurvedic health-care (food advice, herbal medicine, yoga, breathing exercises and daily routines) as well as the component or every-day application of Ayurvedic health care.

### Methods and within-case selection

A case is studied in its natural environment and gives the opportunity to use many sources of data and methods to collect data [17]. In this study a questionnaire was combined with in-depth, semi-structured interviews. The principle of within-case selection followed a strategic logic of maximizing the wealth of the information (not the logic of representativeness) [18].

All persons who attended Ayurvedic health counselling at the present centre from January to May 2014 were asked to participate in the study. As part of the counselling process, questionnaires that are developed by the therapists are used regularly to probe for the client’s background as a tool for diagnosis and to help in the clinical interview. They are filled in by the client before she/he is examined, and are discussed with the therapist after examination. The questionnaires have not been validated for research purposes as they have been developed for the purposes of the counselling process, providing the counsellor with relevant information. The questionnaires adress a variety of background information, including: age, sex, illness; whether they had sought medical care for their illness (yes or no); whether they were satisfied with the medical care (yes or no); whether they were taking any medication for their illness (yes or no); how they experienced their emotional state (classified as good; depressed/anxious; stressed; or other), and whether they practiced any exercise (classified as none, yoga/qi-gong/pilates, or other). The first step of the research project data collection involved collecting these questionnaires after the clients had given written informed consent.

The next step in the research process was to select informants for in-depth interviews. A sum total of 18 persons were selected according to an equal distribution for the codes of ICD-10 illness chapters (described above). A sum total of 18 persons were selected according to an equal distribution for the codes of ICD-10. The distribution of the total pool of 55 participants as well as that of the 18 chosen for interview in accordance with ICD-10 codes is shown in Table 2. The interviews were semi-structured as the aim was to collect “thick descriptions” from the persons by a semi-structured approach. The semi-structure implied open-ended questions that stimulate the respondent to give detailed accounts of their experiences (the interview questions can be found in the appendix). Each interview was conducted by one of two postdoctoral researchers with several years’ experience in qualitative research, in a setting chosen by the informant, in order to provide a secure environment [19]. The interviews varied in length from 40 min to 2 h.

## Analysis

A combination of quantitative and qualitative analysis was utilized. The quantitative analysis gave a backdrop and overall picture of the care-takers’ motives, and it also indicated which specific symtoms came to the fore in the Ayurvedic consultation. The qualitative analysis gave a more nuanced view on the reasons for seeking Ayurvedic health counselling. The same person often had many different health complaints at the same time. This was partly due to the holistic nature of the counselling process, where the person was asked to recount for many aspects of her/his health status.

### Quantitative analysis

The questionnaires were coded and compiled using SPSS analysis. To provide a general picture of the clients’ illness profiles, they were coded according to WHO international classification of diseases (ICD-10) chapters (without sub-classification). Each person was categorized according to her/his major motive for searching Ayurvedic health counselling. This was done according to an interpretation of the major motives of the person, as the persons in many cases reported a complex picture with multiple complaints. This complex picture could be studied in the in-depth interviews that followed.

We performed descriptive analyses on the study participants’ survey responses according to the questionnaire categories described above.

### Qualitative analysis

The interviews were transcribed verbatim and analyzed according to a procedure described by Giorgi [20] and developed by Malterud [18]. Analysis was conducted by two post-doctoral researchers, with experience in medical and humanistic research respectively. The analysis procedure contains four essential steps:

1. Getting a “sense of the whole” in order to arrive at a preliminary organization of the material. The aim in this first step was to get an overall sense of informants’ perspectives and the themes that are relevant to clients in Ayurvedic health counselling.
2. Discrimination of “meaning units” and “coding” them into categories. The aim of this step was to systematically review the material to identify and classify “meaning units” that contain and classify the essential aspects of the interview content.
3. Condensation and abstraction, by comparing and relating the meaning units to each other. This was done according to the respective researchers’ expertise. The interdisciplinary research team could interpret the material by using perpectives from both medical and humanistic sciences.
4. Synthesis and recontextualization. The aim of this final step was to summarise descriptions and develop conceptualisations as a result of a critical/systematic reflection, and to recontextualise the descriptions in relation to the interview material by finding citations that illustrate the essential nuances of each category.

In qualitative research, multiple researchers can strengthen the design of a study by supplementing and contesting each others’statements. In the present study this was achieved through combining the two complementary perspectives of the researchers – one in public health and the other from the psychology of religion. The analysis process conducted in this manner also had the aim to reach saturation, where significantly different accounts were no longer being heard despite interviewing additional participants.

The study has obtained ethical consent from the Stockholm regional research ethics council, with clearance number 2013/2234-31/1.

## Results

### Quantitative results

Between January and May 2014, 59 individuals were asked to participate in the study and 55 persons (93 %) agreed to participate. The participants’ background characteristics, including primary illness (or lack of such), were collected through a questionnaire are shown in the Tables 1 and 2 below. The background characteristics of the participants chosen for interview according to ICD-10 categories were similar to those of the total population, and are shown in Table 1.

**Table 1:** Background characteristics of participants

Characteristic	all (*N* = 55)	Int. (*N* = 18)
Age		
Mean	47	46
Range	27–70	37–70
	% of total	
Sex		
Male	9	6
Female	91	94
Sought health-care for illness?		
Yes	42	56
No	58	44
Satisfied with health-care? (*N* = 22)		
Yes	64	70
No	36	30
Medication?		
Yes	42	56
No	58	44
Emotional state		
Good	53	39
Depression/Anxiety	20	17
Stress	7	6
Unspecific/problematic	20	39
Exercise		
None	7	11
Yoga/Pilates/Qi Gong	56	67
Other	38	22

**Table 2:** Illness panorama of participants, according to ICD-10 classification

Illness	Number	Percent	Int.
None	20	36.4	4
II: Neoplasms	4	7.3	1
III: Diseases of the blood and blood-forming organs, and certain disorders involving the immune mechanism	1	1.8	-
IV: Endocrine, nutritional and metabolic diseases	2	3.6	-
V: Mental and behavioral disorders	1	1.8	1
VI: Diseases of the nervous system	2	3.6	-
IX: Diseases of the circulatory system	4	7.3	1
X: Diseases of the respiratory system	3	5.5	2
XI: Diseases of the digestive system	1	1.8	1
XII: Diseases of the subcutaneous tissue	3	5.5	1
XIII: Diseases of the musculoskeletal system and connective tissue	8	14.5	3
XIV: Diseases of the genitourinary system	2	3.6	1
XVIII: Symptoms, signs and abnormal clinical and laboratory findings, not elsewhere classified	2	3.6	1
XIX: Injury, poisoning, or certain other consequences of external causes	2	3.6	2

Table legend: Int. (Interviewed in qualitative study)

### Qualitative results: motives for seeking Ayurvedic health consultation

The interviewers asked the participants to recount their main motives for seeking Ayurvedic health counselling. Even if all consulations involved the treatment of symtoms, the informants had two principal ways of relating to this. For the first group, the symptoms were the principal motives for engaging in Ayurveda. These informants recounted a number of specific reasons for seeking Ayurvedic health counselling, ranging from more severe illnesses, such as rheumatic pains, cancer, ulcerous colitis, hypothyroidism, and depression. There was also a second group not reporting any serious illnesses. Their principal motives for engaging in Ayurveda were not the symptoms per se, but a striving for harmony and balance in life. The motive could also be a general sense of curiosity and willingness to test “something new”. In these cases, the consultation also involved the treatment of symptoms, but they were not the principal motives for seeking. Furthermore, the symtoms recounted were milder or more diffuse, such as acne, general stress or weight gain.

Also, being helped to find ways to take responsibility of their own health, to gain agency and hope, was an important factor for many, as described by the following informant.

*”In fact, I have an [Ayurvedic] cook book, because then [if I get rheumatic pains again] I will try again, because one can’t just go around in pain. And that’s what feels nice, is that I have something that I can try out, that gives me a sense of hope.” (12/230)*

However, in all groups we could discern a general intention to understand the underlying cause of illness through a holistic view. This was probably a result of the selection process, as this focused people actively seeking Ayurvedic health consulation. The participants can be roughly divided into two groups. One of them endorsed holism in a more general sense; an opinion that conventional health care needs to be complemented with perspectives that emphasize lifestyle, food and diet. The other group endorsed holism in a more strict sense, where the informant actually preferred CAM treatments before conventional medicine. Also, those with a clearly stated holistic view sought to get help that is individually tailored to their specific body constitution and needs.

The following participant expresses a form of individualised holism that had drawn her to Ayurvedic counselling:

*“P: I have also acquainted myself with traditional nutrition and such. But what I like about Ayurveda is that, in part, it integrates the food with advice about yoga and breathing exercises, and also that it is based on that one has a constitution based on these three..*

*I: Doshas?*

*P: Yes, exactly. And depending on who you are, some things will be good for you and other things you should avoid. Or that you should strengthen…*

*I: That it is individualised?*

*P: Yes, that it is individualised, and that there is not one piece of advice that suits everyone.” (19)*

This indicates that participants were familiar with holistic thinking about health issues and had a holistic view on their life problems. Participants shared a sense that illness was caused by an imbalance in a holistic system, and that healing could come out of finding balance. The following participant exemplifies this view by relating her health behaviours to listening to her body, as well as relating to the larger context of seasons.

*“What I really think is neat about Ayurveda is this thing that…it’s not about always doing something static, but that if I need to do a cleansing, […] then I can’t just keep on doing it constantly, but[…] when I’m done with it, then there are other things I should do. I need to follow the seasons. In the summer maybe I can eat other things than I can’t eat in the winter…so I should listen to my body and follow the cycle.” (17)*

As part of this holistic view, there was variety in that some individuals emphasized a strong sense of spirituality, while others did not. This was complicated by the fact that many did not use the concept “spirituality” but still could be said to hold metaphysical or extra-empirical views on their health seeking behavior. According to these individuals, Ayurveda in specific, or CAM and holistic medicine in general is seen as being open to “something more” in life, but this is not necessarily labeled “spirituality”. The following participant defines spirituality as seeing oneself as part of a larger whole.

*“I: You mentioned the word’spiritual’. Is that something you identify yourself with?*

*D: Yes. It’s not about any specific religion or so, but I see it more as a whole. To see oneself as a part of a larger whole.”*

### Contrasting Ayurveda with conventional medicine

Though not specifically asked about their views on conventional medicine, the participants were found to represent either a complementary or an alternative view. Many had health complaints that they sought Ayurveda for, but for which they did not regard conventional, biomedical health-care to be a viable option, even if not all participants had negative experiences with biomedicine. A more negative view was mainly due to concerns about the frequent prescription of medication by biomedical practitioners, combined with concerns about them not addressing the actual root causes of health complaints.

The following citation illustrates a concern about conventional medicine only treating symptoms, instead of the root cause of illness.

*“The reason why I sought Ayurveda was that I have ulcerous colitis, an inflamed bowel, which I have had for several years and I’ve eaten medicine everyday. ne gets a bit tired of just eating medicine,’cause it feels like it just gets rid of the symptoms.” (5)*

The following participants expresses concerns about a lack of holistic view in biomedicine, where knowledge about the impact of food on health is sensed to be lacking.

*“Today’s doctors basically know nothing about food, or even barely about vitamins. They just medicate you instead of making changes in your diet. It’s crazy.” (24)*

The following participant describes being drawn to Ayurveda due to concerns about the unnaturalness of biomedical medication, and contrasts this to a holistic view of “being in a balanced state”.

*”Q: What is it that scares you about taking sleeping pills?*

*A: Well, because it would be so scary not to be able to for example sleep by myself, which is totally natural…I mean, we are born to have daily rhythms and to in some way have destroyed that ability, one’s own process would be very discomforting. They’re such strange medicines that they prescribe, they have such strong adverse effects, so you get scared. But when I’m in a balanced state, then I have no problem sleeping.” (16)*

The following participant also expresses a discontent with what she describes as a lack of a holistic view-point in conventional medicine, mainly regarding environmental factors in illness causation.

*“P: As it was with my eczema, it was horrible, really all over my body. And there was no one [in biomedical health-care] who asked me anything really. Not even what I worked with, in case there could be something in my work environment that I was reacting to. I mean there were no questions whatsoever, they were just “here you have some cortisone”. And then I got some antibiotics because it became so infected, because I had scratched my skin broken, so it had gotten infected. But nothing else.” (3)*

Some participants sought Ayurveda due to an experience of not being helped or believed by biomedical practitioners. Some experienced a discrediting of their symptoms or health problems because they could not be observed through diagnostic tests, and others had sought health-care, but not gotten better despite multiple care efforts. The following participant expresses a disappointment with the lack of preventive measures in conventional medicine, as well as the reliance on what she describes as limited diagnostic tests to detect illness.

*“But what I feel about Ayurveda is that it’s a way to develop oneself, both mentally and physically, to learn to listen to your body’s signals, to know what makes me healthy . It’s a way to keep myself healthy, which is something I feel western medicine doesn’t do whatsoever, but instead, there they just wait until you’re sick. If I go to the doctor and say that I’m not feeling well, they’ll take a blood test. And I have excellent values. I’ve been and taken them, and yes man, I’ve been to one of those health checks, I’m on top, optimal values of everything, even though I’ve been…pretty sick. […].I haven’t felt as well as what those test indicate. And then, I guess either they call it burn-out, or they can’t determine a diagnosis at all…or they can’t decide…I mean, I didn’t get any help. “(17)*

The following participant describes a shortcoming in the diagnosis of her pain condition, where the causal factor was not found despite multiple diagnostic tests. This she expresses led to a sense of frustration and disappointment with the narrow focus on pain relief with painkillers.

*“So I called my family doctor and booked a time at once, and he told me to take pain killers. Then I went to a doctor, and he didn’t know at all what it was. I went home from there just with more painkillers… I took them for two weeks, but it didn’t help at all. Then the pain started spreading to all my muscles, I had pain in all my large muscles, arms, legs, so I went back. The doctor started examining me, they took a whole bunch of tests, X-ray, blood tests, but they couldn’t find anything! The only thing they could find was that I had an inflammation in my body, but not at all what had caused it. So I was just supposed to take painkillers, but they didn’t know the reason. In the end my doctor just sad “I can’t help you, you should maybe seek alternative medicine”.” (18)*

Yet another participant recounted being disappointed with her symptoms having been psychologised, where she herself sensed that they instead had a physical basis.

*“So I tried to find alternatives to feel better. Especially since the doctors didn’t believe me, so I was forced to find other ways. They thought it was something psychological, but I could feel it myself that it was physical, so I wanted to find others who would believe me. So I wanted to find something alternative.” (33)*

One participant sought Ayurveda even though she was satisfied with biomedical health-care, but wanted to use Ayurveda as a complement.

*“I: And how did your doctor react to that you had been to an Ayurvedic consultation?”*

*P: As said, she was really good. I think because it was like that from the start, because I wanted to try to do as much as possible to give my body the best possible circumstances,and there was no speak of that it would only be the cytotoxins that would take away the cancer. So I believe very much in the combination. That it doesn’t need to be just one or the other, but that one can combine.” (19)*

Other participants recounted that in case of a severe illness she would go to a biomedical doctor, but that Ayurveda could be used in combination with conventional care.

*“Ayurveda has existed for thousands of years in comparison with our modern medicine which has existed just maybe for a hundred. So I think that it’s a complement. If I would get a serious illness, then I think I would complement with both parts. And I think that…I don’t see them as two separate worlds, but that they can work together.“(15)*

### Experience of diagnosis at the Ayurvedic consultation

The main means of diagnostics at the consultation were pulse reading, combined with anamnesis. The interviewers asked the informants to recount for their experiences from the process of diagnosis. Participants expressed a sense of being impressed by the accuracy of the diagnosis, and by that the consultant could find out so many things through pulse reading. The following participant recounted being impressed in general by the consultant picking out things that she had not been told about:

*“I felt secure [at the consultation]. I was impressed by her knowledge. As said, I had no expectations, I was mainly just curious. But she picked out one thing after the other that I hadn’t told her about, and by that I must say, I was very impressed.” (10)*

The following participant compared the pulse reading to being to a “clairvoyant”.

*“So the first time I went there, I had to fill in that form […] so I filled it in. She didn’t ask anything, I didn’t say anything, I just filled it in. And then she felt my pulse rate, well, it’s usually low….so she felt like they usually do, for a long time, for 10–15 min. And then she started telling me! Everything she told me was correct, I mean, it was like being to a clairvoyant or something. I’ve never been to one, so I don’t know, but that’s how it felt. Everything, about 95 % of what she said was precisely correct!” (18)*

In more specific terms, the following two participants listed specific ailments that could be found through pulse reading.

*I: OK, interesting. Can you give examples of what it was that was correct?*

*P: Well, first of all that she could feel that I had an inflammation in my body, and then also other things about my personality and so. I was so impressed “(18)*

*“I was surprised [by the consultation]. Because what she said was correct. Just after some minutes. She felt my pulse and then she asked me to write down what problems I had. It was my stomach, and my joints, and asthma. And she figured out all of it without even seeing what I wrote. I was impressed.” (29)*

Other participants, however, recounted that the diagnosis was not completely consistent with their own experience or with medical diagnosis. One participant did not relate to being depressed, which is what the consultant suggested in regard to the pulse reading.

*“But one thing that she said, which she couldn’t feel in my pulse, was whether I was depressed. And that I didn’t really relate with. Or, well, the autumn had been very tough, and the spring. So I haven’t really been myself, but I wouldn’t label it depression.“(9)*

Another participant recounted that the consultant failed to pick out a severe problem in her heart through the pulse reading.

*“She said I have lots of ”ama”, which was accumulated in my liver so that it didn’t work properly, which caused my bad digestion. And that didn’t seem too strange. But then on the other hand, I had some severe problems with my heart, and that she couldn’t feel.” (12)*

### Experienced effects from Ayurvedic consultation

The interviewers asked the informants to recount for their experienced effects from the advice that they had followed. Mostly all accounts of effects were positive, but varied in degree and type. They included concrete relief of physical symptoms, such as reduced exzema, pain, or digestive problems, as well as more diffuse effects such as being more aware of one’s body’s signals, or stress reduction. Even if all participants reported some effects, some did not recount the effects as being strong. Some persons also reported the effects to decrease with time and motivation. The following participant expressed concrete effects in terms of her stomach pain disappearing.

*“So I quit eating meat. And I can say that I had had chronic stomach pains for 2 years, it was like a tooth pain, but in my stomach. […] But in the end I got this advice to quit eating meat, and in two weeks my stomach was symptom-free. “(17)*

This participant recounted amazement with the cessation of her rheumatic pains.

*“I had muscle rheumatism..[…] So I started doing as she said, I began with excluding everything…and you know, within a week I was totally pain-free.*

*I: Oh, so the pain got so much better?*

*P: Yes, I haven’t needed to eat medication since then!” (18)*

Yet another participant described discontinuing her asthma medication, despite which her health had improved.

*“I received a mixture, because I have had asthma since I was small and have used an inhalator for many, many years. I have stopped using it now. Maybe half a month ago. It feels nice. I am very sensitive. I easily catch a cold and if I do it doesn’t stop there, but I become very ill. I get fever and bronchitis or an inflammation, and have to lie in bed with a cough for half a month or up to two months. And I’m totally finished. So it helped actually. During this period when I’ve used these herbs I haven’t been ill.” (29)*

And yet another desribed the disappearance of her exzema in two weeks.

*”P: I’ll try Ayurveda, I thought. And (my exzema which covered my whole body) got better.*

*I: It did?*

*P: Yes, I mean, in two weeks.“(3)*

In terms of mental effects, the following participant described her thoughts as becoming more gathered and her sleep improving.

*”I had more scattered thoughts before, but I sleep much better today and my thoughts are much more gathered. “(11)*

Examples of more diffuse, and less concrete effects are given below, for example in terms of a greater acceptance of life circumstances, as well as awareness of the need to rest and take breaks.

*”I: Has Ayurveda changed your life in any way?*

*P: Yes, I have started to realise that it’s important to take breaks. I always try to fit it into my day, either to do some breathing exercises or some yoga. Sometimes I do it in the evening when the children have gone to sleep, but I always try to fit it into my day. [….And that makes me] feel a sense of that…I accept everything as it is, and not striving somewhere else all the time.” (7)*

Even though all informants reported some form of effect from Ayurveda, some participants did not experience strong effects at all. The following participant explained that she thought the effects will take a longer time to manifest.

*“The other advice I got, it’s like they say, that Ayurveda is a bit more long-term, that it’s not a quick fix that you do in a week. It’s about keeping at it for a long time. So I haven’t seen any clear effects from the advice yet.” (9)*

### Applying the advice in everyday life

When asked about their attitudes on the recommendations given in the consultation many of the participants thought that the advice they obtained was extensive. Some thought that it was even unrealistically so. There was also an evident differences between participants with regard to following the advice, where some described themselves as very motivated, while others found it overwhelming. The following participant found the extent of advice unrealistic within the bounds of contemporary society.

*“But actually, it’s quite difficult these days, in this society, to apply everything [that is recommended at a consultation]. I mean, if one would really listen to one’s body and spirit, then I think one would just sit down and stay put for a couple of years.” (16)*

However, all did not equivocally share this view. The following participant expressed that the advice in fact consisted of quite simple things:

*“Q: What advice did she give you?*

*A: Well, it was mainly about my diet. […]Very simple things, mainly about food. (15)*

The following participant, who was very sick, said that she didn’t find the energy to follow the advice because of their condition, even if she had liked to.

*The thing I felt, about this advice, is that it was a lot of things that I would have liked to follow, but I didn’t have the energy, or the possibility. It was just too much, combined with everything else, when I could barely get out of bed on some days. When I was staying at home and my husband went off to work at 7.15 in the morning, there was no possibility for me to get up myself and make these juices, and with everything that needs to be bought; I didn’t have the energy when I was sick. (19)*

On the other hand, others, who did not have major health complaints, had a tendency not to follow the advice or to “pick and chose” and follow the parts of the recommendations that they thought were most suitable. Those who seemed to have the easiest time following advice, despite their perceived complexity, were the ones who experienced relatively concrete results, or the ones whose advice was relatively simple.

### Relation to other alternative medicine and health concepts

The participants were asked about their main avenues into CAM and specifically which aspects of Ayurveda that were in focus for their interest. Among the most common were yoga practice and interest in health foods and diets. To relate Ayurveda with yoga in this way is typical for Western receptions of modern globalized Ayurveda. For some, social relations and social networks led them to seek Ayurvedic health consultation through for example recommendations from a friend or a person in a yoga class. For others, books and magazines were influential. Frequently mentioned were Janesh Vaidya’s book *Maten är din medicin* (Food is your medicine) and medical doctor Christina Doctare’s book *Vägen till hälsa* (The Road to Health). Noticeably, common for the recounts was an active approach to ones’ health, where the person was well informed and reflected about their decisions.

The interviewers asked the informants to contrast Ayurveda to the other CAM and dietary approaches. Some of the informants had a vast experience of other CAM methods, while for others, Ayurvedic health consultation was their first point of contact with CAM. A “self help” approach, that includes both holistic health methods and conventional medicine was common among the informants. These informants used chosen aspects of Ayurveda in complement to other health methods. One informant called this “att plocka russinen ur kakan” (cherry-picking). Some of our participants, especially many with more concrete and severe physical ailments, reported positive, and often even dramatic beneficial effects. A number of them expressed also a sense of surprise and wonder over the positive effects, reflecting a lack of prior expectations.

There were also some informants who could be described as more “dedicated” to Ayurveda proper, including the following participant, who found Ayurveda superior, more inclusive, holistic or individually accustomed than anything else they had tried out.

*“I have really tried to get help from all sorts of alternative medicine, and all types of treatment. Zone therapy, kinesiology, homeopathy, you name it. I’ve even taken a course in kinesiology. But for me, the major transformation came with Ayurveda. Because I have knowledge about the body, I have acquainted myself with various alternative methods, I have worked with western medical science […], and I know how to think in medical terms. But for me Ayurveda became a kind of wisdom, an understanding of how everything is linked together.” (17)*

The following participant contrasted Ayurvedic dietary recommendations to other popular diets, describing the Ayurvedic approach as superior due to being individually tailored.

*“One can try out all sorts of diets, there’s GI, and 5:2, and this and that, but I believe more in the [Ayurvedic] approach, where you look more at the individual, and adjust the diet to that.” (19)*

## Discussion

### Discussion of demographics

Our study population demographics correspond to those from other settings in Europe and the USA. With regards to Ayurveda in particular, two studies performed in Germany showed that a vast majority of patients are female, middle aged, and have higher education, and this profile has been found in the USA as well [21, 22]. However, in Sweden, with regards to CAM in general, the gender bias was not as strong – 53 % of CAM users were female – even if most users had higher education (81 %) and were in the age range 30–59 years (56 %) [14]. With regards to the illness panorama of our participants, they correspond relatively well to those found in a study at an Ayurvedic tertiary care hospital in a northern state of India: The common reported morbidities at the study hospital were: Respiratory (10.5 %), neuromuscular (9.5 %), digestive (9.2 %) and circulatory (9.1 %) disorders. The majority of diseases were unclassified [23]. However, that study was among a clinical population, and thus direct comparisons cannot be made. Nevertheless, the illness panorama of our participants was similar: Diseases of the respiratory system (5.5 %), Diseases of the musculoskeletal system and connective tissue (14.5 %), and diseases of the circulatory system (7.3 %) were among the most frequent. In addition, neoplasms (7.3 %), and diseases of the subcutaneous tissue (5.5 %) were among the most common in our study, while diseases of the digestive system were relatively uncommon (1.8 %). Over half of the participants practiced yoga, which through the understanding from the qualitative results seemed to be one of the main pathways into Ayurveda.

The over-representation of females is a widely reported phenomenon in CAM practice in general, and Keshet et al. [24] have recently published a literature review on the reasons for this over-representation, which reveals a picture of multiple contradictions. One aspect discussed in the review is that women’s traditional family roles of caring, and responsibility for the family’s health and emotional well-being lead them toward the holistic milieu in greater numbers than men [25]. Also, CAM methods are predominantly constructed and conceptualized by practitioners as ‘soft’ and ‘feminine’ alternatives to the ‘hard’ scientific practices of biomedicine [26]. With its institutional marginality, lack of politically supported legitimacy, and absence of a solid institutional base, CAM is often associated with the private sphere of familial care, which is historically associated with women [26]. On the other hand, women who become dissatisfied with the roles of wife, mother, and nurturer are also described to use holistic and spiritual practices to develop a new and stronger sense of self that is facilitated by getting in touch with a ‘core self,’ [25]. In this sense, CAM in general, and Ayurveda in particular may also become catalysts for change, resistance to traditional meanings of femininity, and the reconstruction of self-identity [24].

### Discussion of qualitative results

Our participants mainly recounted an interest in Ayurveda that arose due to an interest in holistic and individualised treatment, and understanding and treating the underlying cause of illness or imbalance. According to Heusser [27], patients often seek CAM because they miss certain aspects in conventional medicine. This includes a more empathetic practitioner-patient relationship, a better consideration of individual needs, more participation in decision making, more possibilities for self-activity such as life-style and nutrition changes, more comprehensive or holistic strategies and a better inclusion of psychosocial and spiritual aspects in health care [28]. This is partly reflected in the accounts of our participants, though psychosocial aspects were not often mentioned. Indeed, many of our participants mentioned not wanting to seek conventional care for their ailments, because they didn’t like the idea of pharmaceutical treatment aimed at symptomatic relief alone. According to Bishop [28], beliefs about the importance of holistic and natural treatments reflect an emphasis on treating the whole person, instead of just the symptoms, and using what are seen as “natural” methods or remedies instead of processed medicines [28].

For some of our participants, turning to Ayurveda was not related to the perceived narrow focus of the principles of conventional medical care alone, but also to an experience of failing to be helped or understood within the framework of conventional health-care. For example, some participants had failed to obtain a diagnosis or understanding of the underlying cause of their illness, and experienced this as a major frustration, motivating their turning to Ayurveda. Rhodes and co-workers [29] have previously discussed the power of conventional medical tests to provide either positive experiences, which encourage patients to align with their medical providers or, on the other hand, negative experiences of disconfirmation, which lead to alienation and a continued search for resolution. Issues of validation and legitimation are important for patients with chronic conditions [30]. Some of our participants also described having experienced arrogance from the side of conventional medicine towards regards to alternative and holistic methods of healing, which they found frustrating. Indeed, Rhodes and co-workers [29] found that some users of CAM experience “the system” of conventional medicine as unyielding and uncomprehending, requiring intense personal investment from the patient to arrive at some sort of closure. Also, Heusser and co-workers [27] have described that a perceived lack of humanistic qualities constitutes a major problem in modern medicine, a fact that has been described repeatedly and is now beginning to be discussed also in medical education: Patients are frequently unhappy with medical care because physicians fail to demonstrate humanistic qualities. They call thus for courses in the history of medicine, the medical narrative in literature, bioethics, medicine and art, and spirituality and medicine to train physicians, thus tempering conventional medicine with a humanistic touch [31].

Other studies have described the importance of spiritual aspects for those seeking Ayurvedic medicine. In the Swedish context this is complicated by a radical individualization in regard to existential practices, which involves resistance in labeling oneself “spiritual” as well as “religious”, if the person is not allowed to define this in their own terms. Thus, all kinds of categorizations of existential practices are evaded and are experienced as having negative connotations in the Swedish context [32]. In contrast, Kessler et al. conducted a survey study in Germany, regarding spiritual aspects of Ayurveda [10], and found that frustration with modern medicine is less important in the decision to use Ayurveda than, for example, the inclusion of the spiritual dimension [10]. This, however, did not appear to be true for the vast majority of our participants. Even if some of them did mention a sense of spirituality, this was often triggered by the direct questions on this theme by the researchers. Instead, many expressed what can be labeled “holistic beliefs”, that include disease etiologies that are non-empirical and practices that meet existential dimensions of life. For many informants, their interest in Ayurveda was part of a holistic health practice that structured their whole life world. This is in concordance with recent studies on the so called “holistic milieu” in Sweden (eg Frisk, Höllinger, Åkerbäck 2014). However, Kessler [10] also found, in line with our findings, that while spirituality is seen as a very important aspect which also influences the daily life of patients, the medical dimension of Ayurveda is still seen as the most important one [10].

Our findings were similar to qualitative findings from Germany, reported by Frank and Stollberg [33]: Their participants were described to collect objective data about the progress of their health in response to Ayurvedic treatement, mainly in terms of decreased medicine consumption. Indeed, some of our participants also reported decreased need for medication for eczema, IBS, asthma and muscle rheumatism, and described this as evidence for that the Ayurvedic approach had “worked”. Other participants in our study reported more moderate effects and also a tendency of the effects to decrease with time. More research is needed to discern the specific variables that are involved in reporting effects from Ayurvedic treatment, even if some evidence has already emerged: In double-blind, randomized, placebo controlled pilot study comparing Ayurveda, Methrodextrate, and their combination for rheumatoid arthritis, all 3 treatments were found to be approximately equivalent in efficacy [34]. Also, adverse events in the trial were found to numerically fewer in the Ayurveda-only group [34]. For IBS an observational clinical study reported that frequency of bowel movements, bleeding in stool and abdominal pain were dramatically and significantly reduced by Ayurvedic treatment and general well-being dramatically and significantly improved [35]. Additionally, a systematic review of Ayurveda studies for osteoarthritis showed that Ayurvedic drugs seemed safe and effective. However, several limitations in the quality of clinical research warrant more well-planned, well-conducted and well-published trials to advance the evidence-base for Ayurvedic interventions [36].

Participants also frequently reported a sense of surprise about the diagnostic accuracy of pulse diagnostics. Many participants expressed being impressed by the wealth and accuracy of information that could be captured through this form of diagnosis, describing it often as “everything matched”, or “95 % matched” or “she even found things that hadn’t yet shown up in conventional medical tests”. An Ayurvedic clinical examination includes three diagnostic methods: inspection, interrogation and palpation. Inspection involves observation of the body parts, including skin, hair, eyes, tongue. An understanding of the patient’s medical history, symptoms and psychological and physiological characteristics are covered during the interrogation. Palpation includes pulse, and palpation of body parts (abdomen, skin etc.). However, very little is known about the reliability of Ayurvedic diagnostic methods [37]. Frank and Stollberg [33] report from a qualitative study in Germany, that pulse diagnosis has become particularly prominent in the country, and that patients report this technique as particularly impressive. It is, however, also worth noting that the pulse diagnosis was not experienced as accurate by all of our participants. However, in the light of the vast majority being impressed by it’s accuracy, and of the scarcity of previous well-designed trial, further research is warranted. Our participants, like those in Germany, seemed to experience the pulse-reading as surprisingly accurate, which also can motivate further clinical investigation. We have only found a single study on inter-rater variability in pulse diagnostics, which found it to be weak. However, the authors of that study discussed that the feeble inter-rater variability could be explained by a lack of standardised pulse-taking procedure, proper training and experience [37]. Indeed, there are two schools of pulse diagnosis in Ayurveda, and the training in this diagnostic procedure within the Indian BAMS education is dismal, perhaps partly explaining findings of weak inter-rater variability. The Swedish Ayurvedic centre at which the current study was conducted purports to focus in particular on reviving the tradition of pulse diagnosis, and on extensive training in the procedure beyond the scope of the BAMS curriculum.

The most frequently reported negative aspects were related to that following the complex Ayurvedic advice could be found challenging in the current social-cultural context. Mainly, almost all of the participants said that they did not have time to follow all the advice, or that it was cumbersome due to the various challenges such as illness-related fatigue, lack of availability of the products, or general incoherence with behavioral norms. Other research has also reported challenges to implementing the traditional teachings of Ayurveda, centering on the expectations of the client and the practicality of many of the procedures [8].

Finally, it is worth mentioning that there are a number of challenges in biomedical research on Ayurveda, including epistemological diversity and lack of common vocabulary and classification: For instance, dosha as the fundamental process of biological and psychological organization cannot be represented by any formal term in modern biology or biomedical physiology. The Ayurvedic term Sodhana (literally “purification”), an Ayurvedic process of “cleansing” the body and removing “harmful accumulants”, has no biochemical equivalent. Classical botanical terms such as alcaloids and active ingredients does not match the study of herbs from an Ayurvedic perspective, since Ayurveda prescribes whole plants and polyherbal formulations [38]. Nevertheless, there are also examples of successful clinical double-blind randomized placebo-controlled trials when testing Ayurveda in contrast to conventional treatment [34]. Of course, the challenges faced in Ayurveda research are common to those faced also by other CAM methods that are complex and individualized. Thus, researchers have become aware of the necessity to conduct research focused not only on specific evidence but also on unspecific, contextual or patient-centered aspects [34, 39, 40]. Fønnebø et al*.* have highlighted the gap between published studies showing little or no efficacy and reports of substantial benefit from real life clinical practice [41], and this is because CAM treatments are not researched the way they are practiced at the point of care. Therefore, Walach et al*.* propose a circular model instead of a hierarchical model [42] for evaluation of complex medical systems, pointing out that the hierarchical model is valid only for limited questions of efficacy, especially for regulatory purposes and pharmacological products [43].

## Conclusion

Our participants experienced a number of concrete positive effects from Ayurvedic counselling, which motivates further clinical research. Though the clinical research evidence for Ayurveda as a whole system is scarce, and largely unavailable, some evidence for effectiveness is also emerging, some of which coincide with the experiences expressed by our participants. In summary, this case study shed light on a number of interesting topics of further investigation for clinical research in Ayurveda that also applies to research on other CAM methods.
